# *Finegoldia magna*, an Anaerobic Gram-Positive Bacterium of the Normal Human Microbiota, Induces Inflammation by Activating Neutrophils

**DOI:** 10.3389/fmicb.2020.00065

**Published:** 2020-01-29

**Authors:** Ariane Neumann, Lars Björck, Inga-Maria Frick

**Affiliations:** Division of Infection Medicine, Department of Clinical Sciences, Lund University, Lund, Sweden

**Keywords:** anaerobic Gram-positive cocci, GPAC, neutrophils, inflammation, NETs, CD66b expression, host-pathogen interactions

## Abstract

The Gram-positive anaerobic commensal *Finegoldia magna* colonizes the skin and other non-sterile body surfaces, and is an important opportunistic pathogen. Here we analyzed the effect of *F. magna* on human primary neutrophils. *F. magna* strains ALB8 (expressing protein FAF), 312 (expressing protein L) and 505 (naturally lacking both protein FAF and L) as well as their associated proteins activate neutrophils to release reactive oxygen species, an indication for neutrophil oxidative burst. Co-incubation of neutrophils with the bacteria leads to a strong increase of CD66b surface expression, another indicator for neutrophil activation. Furthermore, all tested stimuli triggered the release of NETs from the activated neutrophils, pointing to a host defense mechanism in response to the tested stimuli. This phenotype is dependent on actin rearrangement, NADPH oxidases and the ERK1/2 pathway. Proteins FAF and L also induced the secretion of several pro-inflammatory neutrophil proteins; HBP, IL-8 and INFγ. This study shows for the first time a direct interaction of *F. magna* with human neutrophils and suggests that the activation of neutrophils plays a role in *F. magna* pathogenesis.

## Introduction

Skin and mucosal surfaces are colonized by a plethora of microorganisms, forming the normal microbiota. Among these commensal anaerobic bacteria Gram-positive anaerobic cocci (GPAC) constitute a considerable group. GPAC normally colonize surfaces of the mouth and skin, as well as the gastrointestinal and urogenital tracts (Finegold et al., 1974; Neut et al., 1985; Murdoch et al., 1994; Murphy and Frick, 2013) and do not induce inflammation in a healthy host. However, in wounds or in immuno-compromised hosts, protective barriers like the skin can be breached (Casqueiro et al., 2012), and commensals may turn into opportunistic pathogens causing infection or inflammatory conditions, such as vaginoses, bone and joint infections as well as soft tissue abscesses (Hansson et al., 1995; Wall et al., 2002; Stephens et al., 2003; Brazier et al., 2008; Dowd et al., 2008; Gontcharova et al., 2010; Soderquist et al., 2017). One of the most commonly found species in these infections is the GPAC *Finegoldia magna* (Bourgault et al., 1980; Murdoch, 1998; Bruggemann et al., 2018). In addition to a genomic analysis of the *F. magna* ATCC strain 29328 (Goto et al., 2008), a high aminopeptidase activity was reported and found to be higher as compared to other GPAC species (Ng et al., 1998). This and reports on *F. magna* expression of other enzymes, like collagenase and gelatinase (Krepel et al., 1992), and of capsule formation (Brook and Walker, 1985), indicate a higher pathogenic potential of *F. magna* compared to other GPAC species. To establish an infection and promote its survival in the host, *F. magna* utilizes several surface-bound or secreted proteins (Karlsson et al., 2007, 2009; Frick et al., 2008; Murphy et al., 2014a, b), such as the virulence factors protein L and FAF (*F. magna* adhesion factor) (Bjorck, 1988; Frick et al., 2008). Both of these proteins are associated with the bacterial surface, but can also be released to the environment. The release of FAF from the bacterial surface is mediated via another virulence factor of *F. magna*, SufA (Karlsson et al., 2007). This subtilisin-like protease is able to degrade host defense proteins LL-37 and MIG/CXCL9 (Karlsson et al., 2009). While FAF, like SufA, is able to neutralize antimicrobial peptides (Frick et al., 2008), protein L interacts with neutrophil-derived S100A8/A9 proteins and blocks their antimicrobial activity (Akerstrom and Bjorck, 2009). Protein L also binds immunoglobulin light chains with high affinity (Bjorck, 1988; Akerstrom and Bjorck, 1989).

Neutrophils are among the first cells arriving to the site of infection. Their mode of action includes the secretion of granular proteins, reactive species and various cytokines as well as the formation of neutrophil extracellular traps (NETs). The formation of NETs is a host defense mechanism (Brinkmann et al., 2004) which can be stimulated by a variety of chemical, bacterial, viral, fungal and parasitic stimuli (von Kockritz-Blickwede and Nizet, 2009), resulting in the release of either nuclear or mitochondrial DNA, associated with histones, myeloperoxidase (MPO), neutrophil elastase (NE) and LL-37 (Urban et al., 2009; von Kockritz-Blickwede and Nizet, 2009).

A direct interaction of *F. magna* and its impact on cells of the immune system has not been studied extensively. Two early studies reported the effects of protein L on human mast cells and basophils, triggering the release of histamine and interleukin (Patella et al., 1990; Genovese et al., 2003). The findings here reveal that *F. magna* interacts with primary human neutrophils, resulting in increased CD66b surface expression, production of ROS (reactive oxygen species), HBP (heparin binding protein) release and NET formation, effects that may contribute to the pathogenicity and virulence of *F. magna*.

## Materials and Methods

### Bacterial Strains and Proteins

The *F. magna* strains ALB8 (expressing protein FAF) and 312 (expressing protein L) were isolated at the Department of Clinical Microbiology, Lund University Hospital, Sweden. *F. magna* strain ALB8 was obtained from a patient suffering from a scrotal abscess, while strain 312 was derived from a patient with vaginal infection. Strain 505, naturally lacking proteins FAF and L (Frick et al., 2008; Akerstrom and Bjorck, 2009), was isolated from urethra (Frick et al., 2008). Expression of protein L has been described previously using binding studies to radio-labeled κ-chains (Bjorck, 1988), protein FAF expression was determined by PCR and Western blot (Frick et al., 2008). Bacteria were grown under strict anaerobic conditions in Todd-Hewitt broth (BD Biosciences, Le Pont de Claix, France) supplemented with 0.5% Tween-80 (TH-T; Sigma-Aldrich, St. Louis, MO, United States) at 37°C. Due to challenging cultivation of *F. magna*, for co-incubation experiments with neutrophils, bacteria were washed, heat-inactivated for 20 min at 80°C and adjusted to 1 × 10^9^ cfu/ml in PBS and stored at −20°C until further usage. The heat-inactivation process does not affect the protein binding ability of the bacteria as reported earlier (de Chateau et al., 1993, 1996). The protein L preparation used throughout this study is a fragment covering four of the five repeated immunoglobulin light chain-binding domains (Kastern et al., 1992). FAF was purified as described earlier (Frick et al., 2008) and endotoxins were removed using High Capacity Endotoxin Removal Spin Columns (Thermo Fisher Scientific, Rockford, IL, United States).

### Neutrophil Isolation

Human primary blood-derived neutrophils were isolated from healthy donors as previously described (Neumann et al., 2018). All subjects gave written informed consent in accordance with the Declaration of Helsinki. Briefly, heparinized blood was layered on top of PolymorphPrep ^TM^ (Axis-Shield, Dundee, United Kingdom) and centrifuged at 470 × *g* for 30 min at room temperature (RT). Erythrocytes were lysed by addition of 5 mL sterile water for 15 sec and neutrophils immediately pH-adjusted with PBS. Lysis was performed twice until the cell pellet appeared white, then neutrophils were resuspended in RPMI 1640 medium (Gibco, Paisley, United Kingdom) and the cell number was counted in a Bürker chamber using trypan blue. Cells were adjusted to 1 × 10^3^ cells/μl, then 50 μl were added in 96-well plates and 100 μl in 48-well plates.

### Measurement of Oxidative Burst

Neutrophils were seeded in a 96-well plate and labeled by adding 100 μl 2,7-dichlorofluorescein diacetate (DCF-DA; Sigma-Aldrich, St. Louis, MO, United States) to a final concentration of 100 μM and incubated for 20 min at 37°C. The cells were centrifuged for 5 min at 370 × *g* and the supernatant was removed. Cells were then incubated with *F. magna* strains ALB8, 312 or 505 at a Multiplicity of Infection (MOI) of 20 or with *F. magna* protein FAF and protein L, 3.8 and 3 μg/ml, respectively, at 37°C 5% CO_2_. RPMI 1640 medium was used as a negative control. Fetal bovine serum (FBS) has been reported to reduce the endogenous ROS production (Mun et al., 2017). Thus, to investigate the effect of ROS scavenging, the same assay was performed in the presence of 2% FBS (Thermo Fisher Scientific, Rockford, IL, United States). Fluorescence was measured every 60 min over 2 h at 485 nm excitation and 520 nm emission with a Victor plate reader (PerkinElmer, Hopkinton, MA, United States).

### Detection of CD66b Expression

Bacterial infection induced by Gram-positive bacteria can be associated with an increased CD66b expression on the neutrophil surface (Schmidt et al., 2012). Thus, neutrophils were adjusted to 1 × 10^6^/ml and incubated for 2 h at 37°C 5% CO_2_ with *F. magna* strains ALB8, 312 or 505 at MOI 20, and with 3.8 μg/ml FAF or with 3 μg/ml protein L. 100 nM fMLP (Sigma-Aldrich, St. Louis, MO, United States) was used as a positive control for neutrophil activation and up-regulation of CD66b surface expression. Cells were then labeled with a CD66b antibody (PerCP-Cy 5.5 clone G10F5; BD Biosciences, Le Pont de Claix, France) for 20 min. Samples were measured using the BD Accuri^TM^ C6 Plus personal flow cytometer (BD Biosciences, Le Pont de Claix, France). Results were plotted as median fluorescence intensity in P2 gate.

### Visualization of NETs

Cells were seeded in a 48-well plate and incubated for 2 h with *F. magna* strains ALB8, 312 or 505 at an MOI of 20 or with *F. magna* proteins FAF and L, 3.8 and 3 μg/ml respectively. RPMI 1640 medium was used as a negative control. After the incubation, cells were fixed with 4% PFA for 5 min at RT and then washed 3 times with 1× PBS. NETs were visualized using an antibody for MPO (1:300, mouse monoclonal, Santa Cruz, Heidelberg, Germany). Primary antibody was diluted in PBS with 1% BSA and 0.3% Triton X-100 and samples were incubated over night at 4°C. Subsequent, samples were washed thrice in PBS and incubated with the secondary antibody (goat anti-mouse A568 1:1000 in PBS, Thermo Fisher Scientific, Rockford, IL, United States) for 1 h at RT in the dark. Finally, samples were embedded in ProLong^®^ Gold Antifade Mountant with DAPI (Thermo Fisher Scientific, Rockford, IL, United States). Slides were analyzed using a Nikon Eclipse TE300 fluorescence microscope with a PlanFluor 40×/0.60 NA objective (Bergman Labora, Lyckeby, Sweden). For each preparation, a minimum of three randomly selected images were acquired and used for quantification of NET-producing cells in ImageJ software. Data were expressed as percentages of NET-releasing nuclei in relation to the total number of cells.

### Inhibition of NET Formation

Neutrophils were seeded in a 96-well plate and incubated with *F. magna* strains ALB8, 312 or 505 at an MOI of 20 or with *F. magna* proteins FAF and L, 3.8 and 3 μg/ml respectively. RPMI 1640 medium was used as a negative control. After 2 h, extracellular DNA was stained with the cell-impermeable dye Sytox Green (Thermo Fisher Scientific, Rockford, IL, United States), which is commonly used to quantify NETs (Carmona-Rivera and Kaplan, 2016). The dye was added to the supernatant of the samples for 5 min and fluorescence was detected at wavelength recommended by the manufacturer 504/523 nm. The following inhibitors were used: actin polymerization inhibitor Cytochalasin D (20 μM; Thermo Fisher Scientific, Rockford, IL, United States), store-operated Ca^2+^ entry inhibitor 2-Aminoethoxydiphenyl borate (2-ABP, 50 μM; Sigma-Aldrich, St. Louis, MO, United States), MAPK/ERK kinase U0126 (50 μM; Sigma-Aldrich, St. Louis, MO, United States) and inhibitor of nitric oxide synthetase diphenyleneiodonium chloride (DPI, 10 μM; Sigma-Aldrich, St. Louis, MO, United States).

### Neutrophil Elastase Activity

For the measurement of NE activity, cells were incubated for 2 h at 37°C with *F. magna* strains ALB8, 312 or 505 at a MOI of 20 or with *F. magna* protein FAF and protein L, 3.8 and 3 μg/ml respectively. After incubation, supernatants were transferred into a new plate containing DQ-elastin (EnzChek^®^ Elastase Assay Kit, Thermo Fisher Scientific, Rockford, IL, United States). This non-fluorescent dye can be cleaved by released enzyme, resulting in a fluorescent signal. Fluorescence intensity was measured according to the manufacturer’s recommendations at 505/515 nm.

### Secretion of Heparin Binding Protein (HBP) and Cytokine Profile

Neutrophils were seeded in a 96-well plate and incubated for 2 h with *F. magna* strains ALB8, 312 or 505 at MOI 20 and with 3.8 μg/ml FAF and 3 μg/ml L at 37°C. Following incubation, the supernatant was collected and stored at −80°C until further usage. Samples were further analyzed using the Heparin Binding Protein EIA kit (HBP ELISA, Axis Shield, Dundee, United Kingdom) or the Bio-Plex Pro^TM^ Human Cytokine 27-plex Assay (Cytokine profile, Bio-Rad, Solna, Sweden).

### Statistical Analysis

Data were analyzed by using GraphPad Prism v7.0 (GraphPad Software, San Diego, CA, United States). Differences between 2 groups were analyzed by using a paired, one-tailed Student *t* test or 1-way ANOVA with the Bonferroni *post hoc* test. Significance is indicated as ^∗^/#*p* ≤ 0.05, ^∗∗^/##*p* ≤ 0.01, ^∗∗∗^/###*p* ≤ 0.001, and ^∗∗∗∗^/####*p* ≤ 0.0001.

## Results

### *F. magna* Triggers Oxidative Burst in Primary Neutrophils

**FIGURE 1:**
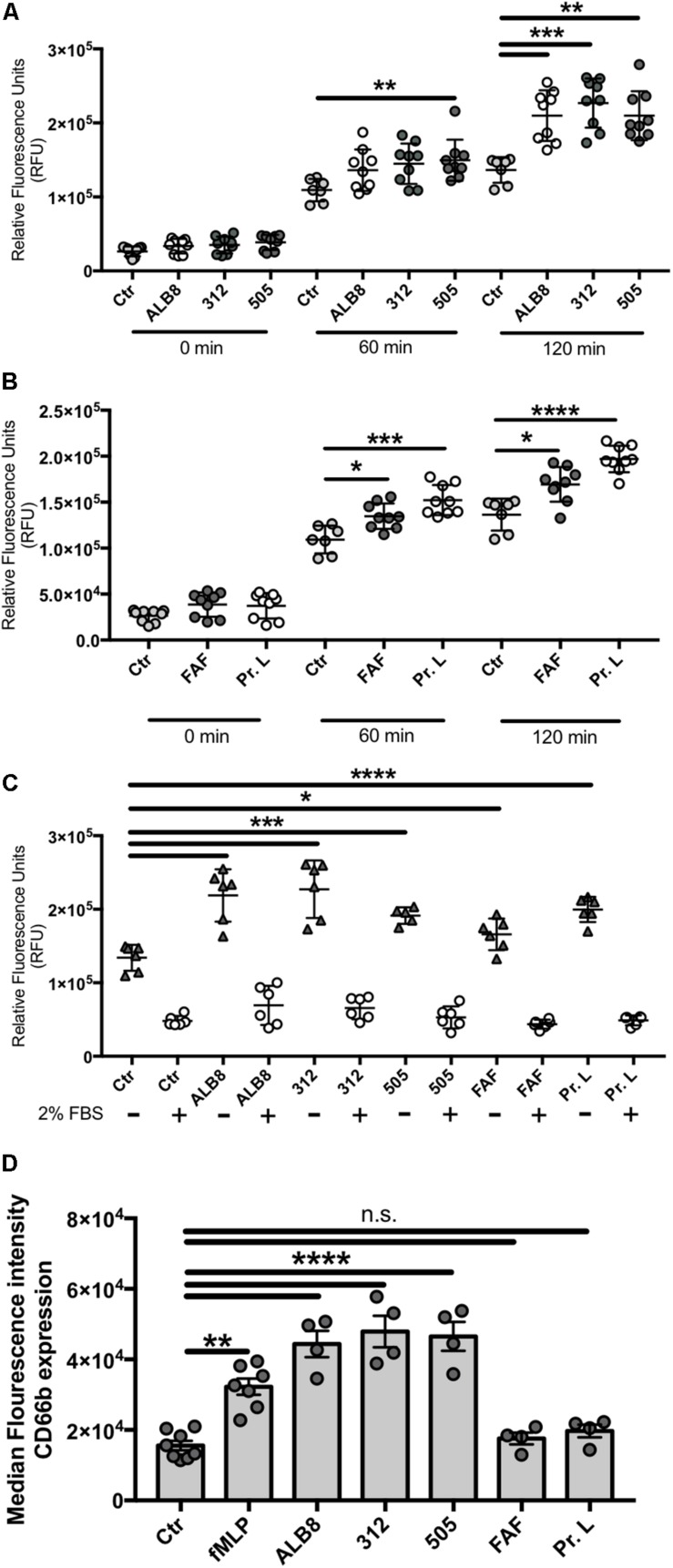
*Finegoldia magna* bacteria and respective proteins activate neutrophils. Neutrophils were incubated with **(A)** heat inactivated *F. magna* strains ALB8, 312 or 505 or with **(B)** purified protein FAF and protein L. Production of ROS was measured every 60 min and is displayed as relative fluorescence units (RFU). **(C)** ROS production was measured in presence and absence of 2% FBS, which reduced all stimuli-triggered ROS production. Control (Ctr) represents unstimulated neutrophils. **(D)** Neutrophils incubated as above were analyzed by flow cytometry. The median fluorescence intensity indicates the CD66b surface expression on neutrophils in response to *F. magna* strains and purified proteins FAF and protein L. All data represent mean ± SEM of 3 independent experiments. *p ≤ 0.05, **p ≤ 0.01, ***p ≤ 0.001, ****p ≤ 0.0001.

Human blood-derived neutrophils and heat-inactivated *F. magna* strains ALB8 (expressing protein FAF), 312 (expressing protein L) or 505 (expressing neither protein FAF nor protein L) were co-incubated 2 h at a MOI of 20. The heat-inactivation step does not affect the protein binding properties of the strains, as reported in other studies (de Chateau et al., 1993, 1996). Neutrophil activation was measured by detection of oxidative burst (Chen and Junger, 2012). All three strains significantly induced ROS production after 60 and 120 min (Figure 1A). Next, neutrophils were incubated with the purified *F. magna* proteins FAF and L and after 60 min a significant activation was obtained with both proteins (Figure 1B). Overall, the degree of neutrophil activation with whole bacteria was slightly higher compared to the activation caused by the proteins after 2 h incubation. Since the production of ROS can be scavenged by serum (Mun et al., 2017), we repeated the 2 h incubation time point in the presence of 2% FBS, and the ROS production induced by all tested stimuli was completely abolished in the presence of FBS (Figure 1C). This result points to an involvement of NADPH oxidases in *F. magna*-mediated neutrophil activation.

### Surface Expression of the Neutrophil Marker CD66b Is Up-Regulated by *F. magna*

The finding that whole *F. magna* bacteria and proteins FAF and L triggered oxidative burst in neutrophils, stimulated us to further investigate *F. magna* influence on neutrophil activation. Increased expression of CD66b is described as a marker of neutrophil activation in Gram-positive bacterial infections (Schmidt et al., 2012). Neutrophils were therefore stimulated with intact *F. magna* bacteria and proteins FAF and L for 2 h, and the expression of the neutrophil surface receptor CD66b was detected using flow cytometry. Compared to un-stimulated neutrophils, the median fluorescence intensity for CD66b increased significantly in the presence of the bacteria (Figure 1D), with values higher than the positive control fMLP. No increase in the median fluorescence intensity was observed when neutrophils were incubated with the purified proteins (Figure 1D).

### NETs Are Released After Incubation With *F. magna* and *F. magna* Proteins

**FIGURE 2:**
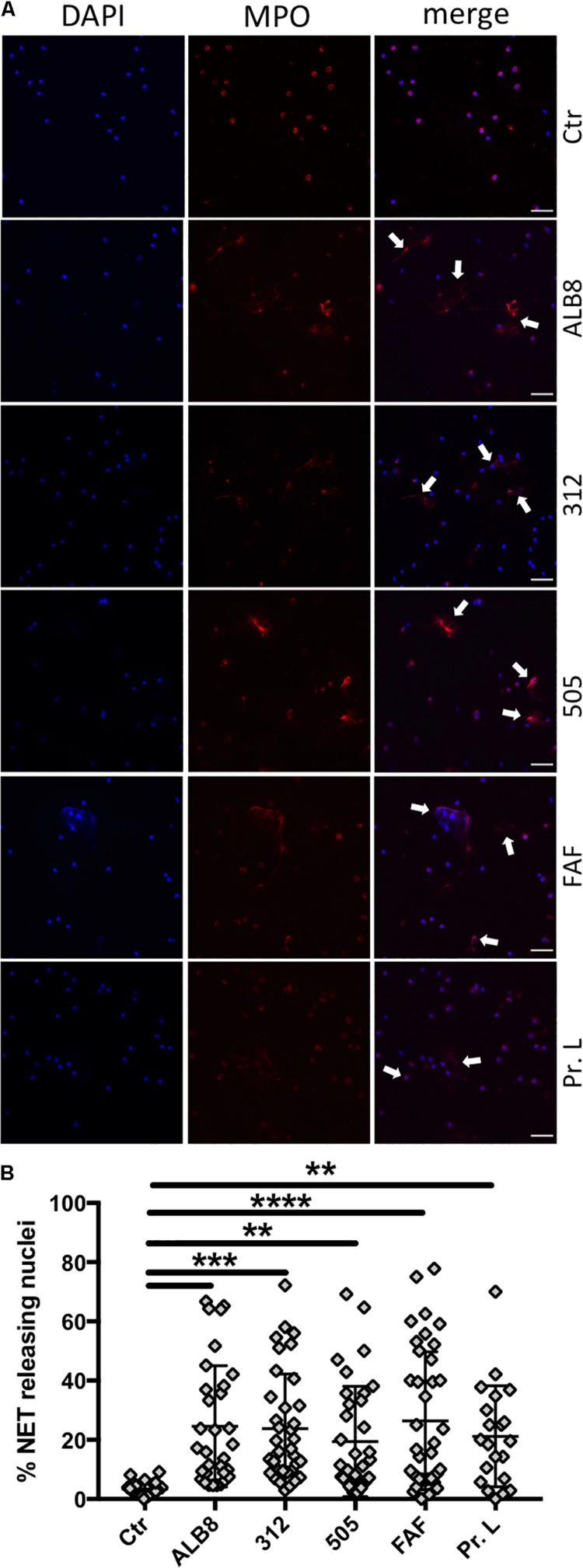
*F. magna* bacteria and proteins induce NET formation. **(A)** Neutrophils were incubated with heat inactivated *F. magna* strains ALB8, 312 or 505 or with purified proteins FAF and L. Control (Ctr) represents unstimulated neutrophils. NETs (white arrows) were visualized with DAPI (blue) and MPO (red), scale bar is 50 μm. **(B)** NET-releasing nuclei were quantified using ImageJ software and are displayed as percentage. Data represent mean ± SEM of 4 independent experiments. **p ≤ 0.01, ***p ≤ 0.001, ****p ≤ 0.0001.

Activated neutrophils have been demonstrated to release their DNA associated with histones and antimicrobial proteins as NETs in response to invading pathogens. Therefore we analyzed whether activation of neutrophils with *F. magna* strains ALB8, 312 or 505, and bacterial protein FAF or protein L, would result in the release of NETs. NETs consist of a DNA meshwork with associated granule proteins, like MPO. We therefore stained the samples for MPO (red) whereas DAPI was used as a counter stain for DNA (blue; Figure 2A). Fluorescence microscopy revealed that MPO is located in granules around the nucleus in un-stimulated neutrophils and DNA in the nuclei is lobulated and condensed (Figure 2A, Ctr). The co-incubation with *F. magna* bacteria and proteins showed that NETs are released, with MPO diffusely binding to DNA fibers (white arrows) and the intensity of DNA staining decreased as previously described (Brinkmann et al., 2012). The architecture and the amount of the NETs varied depending on the stimulus used, with the longest NET structures observed for protein FAF (Figure 2A). Next, NET-releasing nuclei were quantified with ImageJ cell counter software. All stimuli significantly induced the release of NETs after 2 h of co-incubation with neutrophils (Figure 2B). The effect was most pronounced with FAF, however, high donor variability was observed with all stimuli (Figure 2B).

### Analysis of Pathways Involved in *F. magna*-Induced NET Formation

**FIGURE 3:**
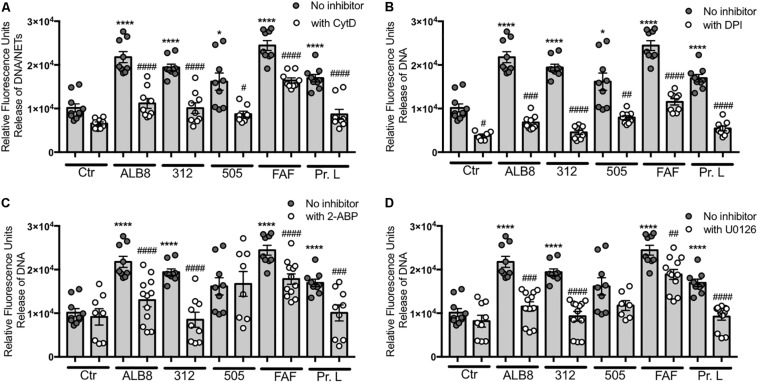
Actin rearrangement and NADPH oxidases are involved in *F. magna*-mediated NET release. Neutrophils were co-incubated with heat inactivated *F. magna* bacteria or proteins FAF and L for 2 h. The release of NETs was blocked by the addition of **(A)** 20 μM Cytochalasin D (CytD), **(B)** 10 μM DPI, **(C)** 50 μM 2-ABP, **(D)** 50 μM U0126. All data represent mean ± SEM of 3–5 independent experiments. *p ≤ 0.05, ****p ≤ 0.0001 to Ctr; #*p* ≤ 0.05, ##*p* ≤ 0.01, ###*p* ≤ 0.001, ####*p* ≤ 0.0001 to stimuli without inhibitor.

Next, we investigated pathways involved in *F. magna*-induced NET formation. For this assay, extracellular DNA was stained with the membrane-impermanent dye Sytox Green^TM^, which can be used as a NET quantification method (Carmona-Rivera and Kaplan, 2016). Cytochalasin D is commonly used as an inhibitor for phagocytosis (Ribes et al., 2010). However, it has also been reported that actin rearrangement is important for NET release (Neeli et al., 2009). We found that in the NET release mediated by both *F. magna* bacteria and the purified proteins, actin rearrangement played a significant role. The effect is most pronounced with bacterial strains ALB8 and 312 and proteins L and FAF (Figure 3A), where the NET formation is reduced in the presence of cytochalasin D to levels of spontaneous NET release. The addition of FBS during *F. magna* incubation with neutrophils resulted in blocking of NADPH oxidases and dampening of neutrophil activation (Figure 1C). The neutrophil NADPH oxidase inhibitor DPI significantly reduced the NET release upon *F. magna* stimulation (Figure 3B), further emphasizing the role of NADPH oxidases in neutrophil activation. Again the strongest reduction was seen for NET release mediated by the bacterial strain 312 and protein L (Figure 3B). DPI also reduced the spontaneous release of NETs (Figure 3B). Furthermore, manipulating the release of intracellular calcium ions (Ca^2+^) using 2-ABP, demonstrated that the release of NETs, triggered by all stimuli, was significantly reduced, except for strain 505 (Figure 3C). The ERK 1/2 pathway was previously described to be involved in NET formation (Munoz-Caro et al., 2015), and a common inhibitor of this pathway is U0126 (Malemud et al., 2015). In presence of this inhibitor, NET formation was reduced to control levels in the case of *F. magna* strain 312 and protein L, and significantly reduced when stimulated with strain ALB8 and protein FAF (Figure 3D). No effect of this inhibitor was detected when cells were incubated with *F. magna* strain 505 (Figure 3D).

### *F. magna* Protein L Induce Secretion of Secretory Vesicles but Not Azurophilic Granules

**FIGURE 4:**
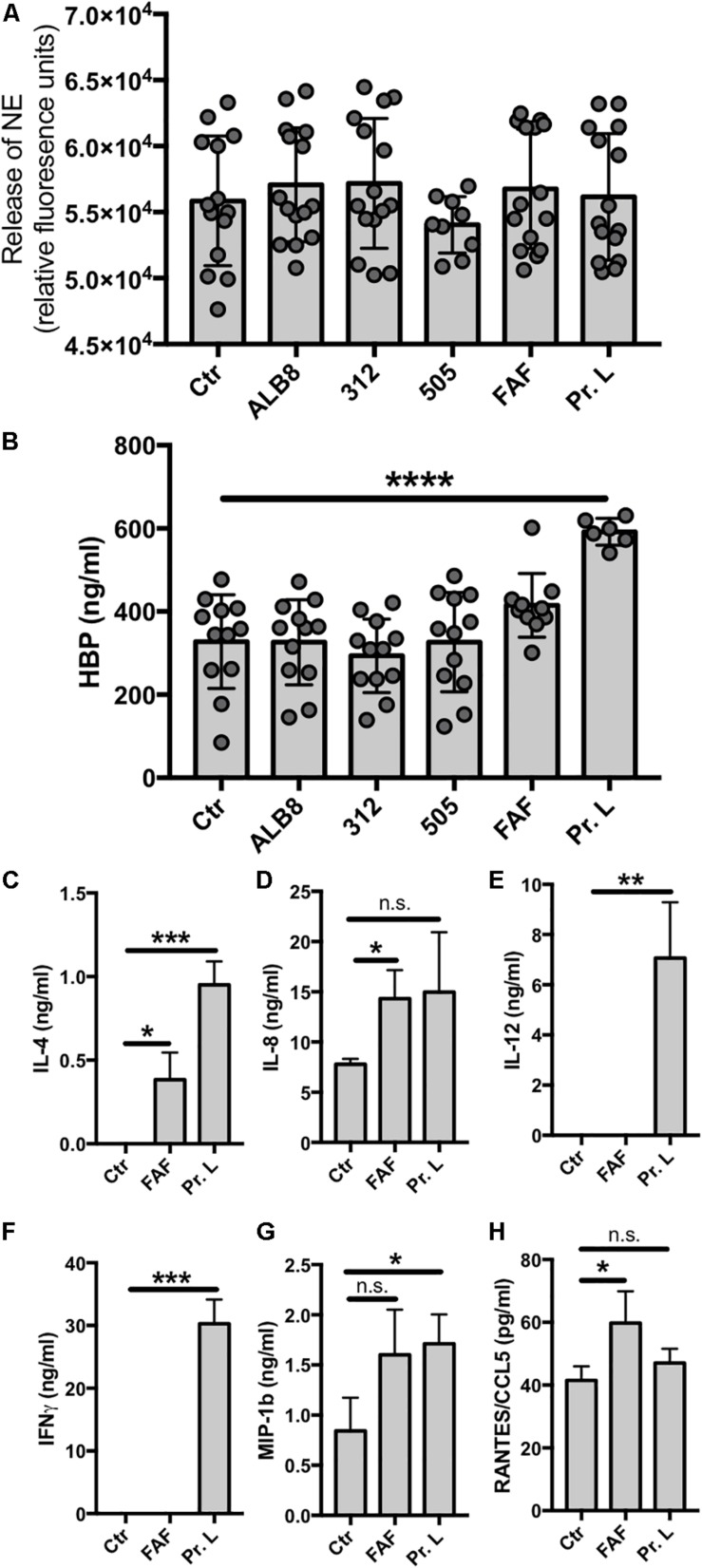
Protein L triggers HBP and cytokine release. **(A–H)** Neutrophils were incubated with *F. magna* bacteria or their proteins for 2 h and supernatants were collected for **(A)** NE enzyme activity, **(B)** HPB release or **(C–H)** cytokine profiling. **(A)** Enzyme activity was measured using NE EnzChek assay kit. None of the used stimuli triggered enzyme activity. **(B)** Protein L significantly induced the release of HBP. Whole bacteria and protein F had no effect on the HBP release. **(C–H)** Cytokine profile was determined after co-incubation of neutrophils with *F. magna*-derived proteins FAF (3.8 μg/ml) or protein L (3 μg/ml) for 2 h. **(C)** IL-4, **(D)** IL-8, **(E)** IL-12, F) INFγ, **(G)** MIP-1b and **(H)** RANTES/CCL5. Data represent mean ± SEM of 3 independent experiments. *p ≤ 0.05, **p ≤ 0.01, ***p ≤ 0.001, ****p ≤ 0.0001.

Both *F. magna* proteins and the bacteria themselves triggered activation of neutrophils resulting in oxidative burst and release of NETs. We therefore investigated the impact of *F. magna* bacteria and proteins on neutrophil degranulation and release of NE and HBP. Recently, NE was suggested as a biomarker for bacterial infection in patients with COPD (Thulborn et al., 2019) and increased levels of HBP secretion have been associated with sepsis (Herwald et al., 2004; Fisher and Linder, 2017). Neither the incubation of neutrophils with intact *F. magna* bacteria nor proteins resulted in an elevated NE enzyme activity (Figure 4A). Interestingly, protein L, significantly triggered the release of HBP (Figure 4B). The secretion of HBP might facilitate the release of pro-inflammatory cytokines from the neutrophils. Thus, in a final experiment, primary neutrophils were co-incubated with FAF or protein L for 2 h and the cytokine profile was analyzed. The co-incubation with FAF led to a significant increase in secretion of IL-4, IL-8 and RANTES/CCL5 (Figures 4C,D,H). Protein L significantly triggered the release of IL-4, IL-12, IFNγ and MIP-1b (Figures 4C,E–G).

## Discussion

*Finegoldia magna* is one of the most frequently isolated GPAC species in clinical specimen. It is found in patients with soft tissue abscesses, acute wound infections, vaginoses, septic arthritis and endocarditis (Murphy and Frick, 2013), and the bacteria are also prevalent in chronic wounds (Hansson et al., 1995; Dowd et al., 2008). Due to challenges in cultivation of *F. magna* and difficulties to obtain good-quality anaerobic specimens, their incidence in clinical infections are most likely underestimated (Murphy and Frick, 2013).

The *F. magna* strains ALB8 (expressing protein FAF), 312 (expressing protein L) and 505 (naturally lacking both protein FAF and protein L), as well as the purified proteins FAF and L, were found to activate human blood-derived neutrophils resulting in oxidative burst. Neutrophils express a multitude of cell surface receptor, which are responsible for downstream signaling during inflammation or infection (Futosi et al., 2013). Transmigration of neutrophils to the site of infection is often accompanied by an up-or down-regulation of those surface markers, such as CD66b (Schmidt et al., 2012). *F. magna* bacteria caused an up-regulation of CD66b, which further suggests that activation of the neutrophils occurred. Pathogen-associated molecular patterns (PAMPs) like the *F. magna* proteins might only be able to activate certain pattern recognition receptors, to efficiently activate the immune system (Brinkmann et al., 2012). This might explain why the co-incubation with only protein FAF or L did not result in up-regulation of CD66b.

Various stimuli can facilitate the formation of NETs utilizing different pathways, as recently demonstrated by Kenny and colleagues (Kenny et al., 2017). Our findings also suggest that *F. magna* bacteria and their proteins employ different pathways to trigger release of NETs, such as actin rearrangement or NADPH oxidases. Additionally, the architecture of the NETs appears multi-faceted when stimulated by e.g., *F. magna* bacteria compared to the proteins FAF and L. Proteomic analysis of NET induced by different stimuli proposed even specific protein composition depending on the stimulus (Petretto et al., 2019). As discussed by Brinkmann et al. (2012) the amount of NETs formed might alter depending on the type of stimulus. Also donor variations could play an important role in the quantity of released NETs (Brinkmann et al., 2012), which is supported by our experiments as shown in Figure 2B. The application of diverse inhibitors in the present study and their effect on the NET formation mediated by the various stimuli further indicates the involvement of distinctive pathways for each stimulus.

Apart from being associated with the bacterial surface, FAF is released by SufA, a subtilisin-like protease, expressed by *F. magna* (Frick et al., 2008). Due to its location on the bacterial surface and its α-helical coiled-coil structure, FAF shows similarities to M proteins of *Streptococcus pyogenes* (Murphy and Frick, 2013). Like FAF, M protein of serotype 1 is also released from the bacterial surface (Berge and Bjorck, 1995), and M1 protein in complex with fibrinogen has been shown to facilitate the formation of NETs (Oehmcke et al., 2009). The release of NETs triggered by FAF and FAF-expressing *F. magna* strain ALB8 further underlines the similarity to M1 protein. Interestingly, in cases of chronic otitis media, bacteria use the neutrophil-derived meshwork to replicate and hide from antibiotic treatment as well as neutrophil-mediated killing (Thornton et al., 2013). Since *F. magna* also has been reported to form biofilms (Murphy and Frick, 2013), the induction of NETs might contribute to this phenotype protecting the bacteria from clearing by the host.

Over 90% of all *F. magna* strains express FAF, which mediates binding to different layers of the skin (Murphy et al., 2014b). Once *F. magna* passes through this protective barrier of the host, the bacteria have to deal with host responses. Neutrophils arrive early to the site of an infection and play, together with antimicrobial peptides, important roles in innate immunity. The formation of NETs as a host defense mechanism (Brinkmann et al., 2004) results in the release of DNA associated with histones and LL-37 among other molecules (Urban et al., 2009; von Kockritz-Blickwede and Nizet, 2009). Histones and LL-37 are in turn antimicrobial against a wide range of bacteria, a property that FAF is counteracting by binding and neutralizing their actions (Frick et al., 2008; Murphy et al., 2014a). In addition, FAF has been shown to inhibit several other antimicrobial peptides (Karlsson et al., 2009; Murphy and Frick, 2013).

About 10% of *F. magna* strains express protein L (Bjorck, 1988). Similarly to FAF, protein L is also released into the medium (Bjorck, 1988; Kastern et al., 1992), where it activates mast cells to release histamine (Patella et al., 1990). The interaction of protein L with human mast cell progenitor FcεRI^+^ cells leads to the synthesis and release of IL-4 and IL-13 (Genovese et al., 2003), and here we find that release of IL-4 from neutrophils is also triggered by protein L. Recently, protein L was hypothesized to be involved in a fatal case of monomicrobial *F. magna* bacteremia, causing toxic shock-like symptoms in a patient (Rosenthal et al., 2012). Protein L also interacts with neutrophil proteins S100A8 and S100A9, thereby protecting the bacteria from the neutrophil-mediated killing (Akerstrom and Bjorck, 2009). Protein L is a B cell super-antigen, which may also contribute to *F. magna* pathogenesis and virulence (Patella et al., 1990; Genovese et al., 2000, 2003; Viau et al., 2004; Anderson et al., 2012). In the present study we find that the interaction between protein L and neutrophils causes a significant secretion of HBP, a protein that induces vascular leakage (Gautam et al., 2001). Human serum albumin is released in case of vascular leakage, which promotes the growth of *F. magna* (de Chateau et al., 1996). This finding further underlines the pro-inflammatory property of protein L. Protein L only triggered the release of HBP, but not NE suggesting that secretory vesicles, but not azurophilic granules are affected.

The exposure of epidermal keratinocytes to *F. magna* resulted in expression of antimicrobial peptides and pro-inflammatory cytokines (Zeeuwen et al., 2017). The present investigation shows that proteins FAF and L both cause cytokine secretion also from primary human neutrophils. IL-4 activates cytoskeletal rearrangements (Girard et al., 1997), which is involved in NET release mediated by FAF and protein L. Additionally, cytoskeletal rearrangements delay apoptosis (Girard et al., 1997), which might facilitate the formation of NETs detected within 1–2 h after neutrophil co-incubation with *F. magna*. A positive feed-forward loop can be achieved by the facilitated secretion of IL-8, mediated by both proteins, since IL-8 itself is a potent inducer of NET formation (Keshari et al., 2012). Another inducer of NETosis might be RANTES/CCL5, which in complex with CXCL4 (platelet factor 4) triggers the formation of NETs (Rossaint et al., 2014). Neutrophils release IFNγ in response to IL-12 secretion (Ethuin et al., 2004), and excessive release of IL-12 and INFγ has been suggested to impair the response to bacterial infection (Zhang and Starnbach, 2015). In the present study we found that protein L significantly triggered the release of both IL-12 and IFNγ. Finally, neutrophils transmigrating to the site of infections have been reported to secrete macrophage inflammatory protein-1 beta (MIP-1β) in order to attract dendritic cells (Chiba et al., 2004). Protein L induced the release of MIP-1β, indicating that neutrophils respond to *F. magna* proteins by means of chemo-attracting other immune cells to the site of infection.

To our knowledge the present work represents the first study of the interaction between the significant GPAC species *F. magna* and human blood derived neutrophils. The results demonstrate that *F. magna* and its soluble proteins FAF and L activate neutrophils and induce a pro-inflammatory response. This and previous findings showing that FAF and protein L also block the effect of antibacterial peptides/proteins, indicates that the combination of these mechanisms adds selective advantages to *F. magna*, which may also increase the pathogenic potential of this member of the normal microbiota.

## Data Availability Statement

All datasets generated for this study are included in the article/supplementary material.

## Ethics Statement

The studies involving human participants were reviewed and approved by the Lund University Ethics Committee (approval 2008/657). The patients/participants provided their written informed consent to participate in this study.

## Author Contributions

AN designed the study, performed the experiments, analyzed the data, and wrote the manuscript. LB analyzed the data and wrote the manuscript. I-MF purified the proteins, analyzed the data, and wrote the manuscript. All authors approved the submitted version of the manuscript.

## Conflict of Interest

The authors declare that the research was conducted in the absence of any commercial or financial relationships that could be construed as a potential conflict of interest.
